# *De Novo* Genome Assemblies for Three North American Bumble Bee Species: *Bombus bifarius*, *Bombus vancouverensis*, and *Bombus vosnesenskii*

**DOI:** 10.1534/g3.120.401437

**Published:** 2020-06-25

**Authors:** Sam D. Heraghty, John M. Sutton, Meaghan L. Pimsler, Janna L. Fierst, James P. Strange, Jeffrey D. Lozier

**Affiliations:** * Department of Biological Sciences, The University of Alabama, Tuscaloosa, AL; † Department of Entomology, The Ohio State University, Columbus, OH

**Keywords:** *Bombus bifarius*, *Bombus vancouverensis*, *Bombus vosnesenskii*, Illumina, Oxford Nanopore Technologies, hybrid assembly, MaSuRCA

## Abstract

Bumble bees are ecologically and economically important insect pollinators. Three abundant and widespread species in western North America, *Bombus bifarius*, *Bombus vancouverensis*, and *Bombus vosnesenskii*, have been the focus of substantial research relating to diverse aspects of bumble bee ecology and evolutionary biology. We present *de novo* genome assemblies for each of the three species using hybrid assembly of Illumina and Oxford Nanopore Technologies sequences. All three assemblies are of high quality with large N50s (> 2.2 Mb), BUSCO scores indicating > 98% complete genes, and annotations producing 13,325 – 13,687 genes, comparing favorably with other bee genomes. Analysis of synteny against the most complete bumble bee genome, *Bombus terrestris*, reveals a high degree of collinearity. These genomes should provide a valuable resource for addressing questions relating to functional genomics and evolutionary biology in these species.

Bumble bees (Hymenoptera: Apidae: *Bombus*) are a widespread and iconic pollinator genus of approximately 250 species globally (Cameron *et al.* 2007; Cameron and Sadd 2020). Bumble bees provide economic and ecological benefits through their pollination services (Greenleaf and Kremen 2006; Velthuis and van Doorn 2006) and have recently generated significant public interest because, like many pollinators, numerous species have undergone rapid declines across the globe (Cameron *et al.* 2011; Cameron and Sadd 2020). Bumble bees also have a long history of study with respect to adaptations in traits like thermoregulation, flight biomechanics, host-parasite interactions, and evolution of sociality, and the advent of genomic tools has greatly accelerated our understanding of their biology (Woodard *et al.* 2015; Lozier and Zayed 2016). However, there remain a limited number of genetic resources to study such adaptive traits and help conservation efforts, with only two annotated reference genomes available on the National Center for Biotechnology Information (NCBI) for *B. terrestris* and *B. impatiens* (Sadd *et al.* 2015) and one additional genome published for *B. terricola* (Kent *et al.* 2018). Species-specific reference genomes can be valuable for addressing population genetic questions about novel targets of selection, comparing structural genetic variation within and between species, and more accurate functional genomics studies including transcriptomics (RNAseq) or epigenetics (*e.g.*, bisulfite sequencing) (Fuentes-Pardo and Ruzzante 2017). Additional genomic resources could also prove to be useful from a conservation standpoint, including better understanding of species and population-specific genetic variation (Allendorf 2017).

We present three new assemblies for species within the *Bombus* subgenus *Pyrobombus* (Cameron *et al.* 2007; Williams *et al.* 2008): *B. vosnesenskii* Radowski, *B. bifarius* Cresson, and the recently re-described *B. vancouverensis* Cresson (subspecies *nearcticus*) (Ghisbain *et al.* 2020)

**Figure 1:**
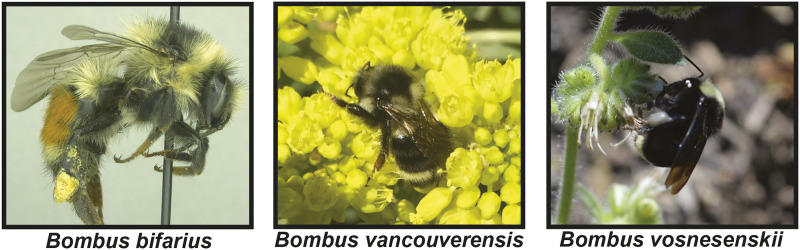
Three focal species used for genome assembly.

(Figure 1). These species are among the most common bumble bees in western North America (Koch *et al.* 2012) and have been the focus of recent research that includes studies investigating gene flow, foraging range, and genetic diversity in natural and agricultural systems (Lozier *et al.* 2011, 2016; Rao and Strange 2012; Jha and Kremen 2013; Jha 2015; Geib *et al.* 2015; Jackson *et al.* 2018; Mola *et al.* 2020), climate-driven local adaptation across spatial-environmental gradients (Jackson *et al.* 2020), and color pattern variation and speciation (Pimsler *et al.* 2017; Ghisbain *et al.* 2020).

We employ a combination of long-read (Oxford Nanopore Technologies, ONT, Oxford, UK) and short-read (Illumina, San Diego, CA) sequencing technologies to produce high-quality hybrid assemblies for each of the three species. The assemblies produced here perform well compared to other published *Bombus* genomes and will provide a valuable resource as reference genomes for research into comparative and evolutionary genetics of *Bombus*.

## Materials and Methods

### DNA Extraction and Sequencing

Individuals used for genome sequencing include a field-collected female *B. vancouverensis nearcticus* worker (JDL1245, diploid) from Sequoia National Park, CA, a field-collected male *B. bifarius* (JDL3187, haploid) from Arapaho National Forest, CO, and two males from a laboratory *B. vosnesenskii* colony (JDL3184-5, both haploid) reared at the USDA-ARS Pollinating Insects Research Unit in Logan, UT (queen from Emigrant Lake, OR) (Table 1). Whole genomic DNA was extracted from the thorax, and in some cases include abdominal tissue if additional DNA was required for Illumina sequencing, using the Qiagen (Valencia, CA) MagAttract High Molecular Weight kit or DNeasy Blood and Tissue kit (for Illumina sequencing only). Extractions were cleaned using Ampure XP beads (Beckman-Coulter, Pasadena, CA) at a ∼0.4x concentration to remove small DNA fragments and improve purity.

**Table 1:** Summary of sample collection information

Species	Sample	Source Locality	Latitude	Longitude	Elevation (m)
*B. bifarius*	JDL3187	Boulder County, Colorado, US	39.940	−105.560	2,760
*B. vancouverensis*	JDL1245	Tulare County, California, US	36.597	−118.736	2,214
*B. vosnesenskii*	JDL3184	Jackson County, Oregon, US	42.152	−122.621	685
	JDL3185	Jackson County, Oregon, US	42.152	−122.621	685

Long read sequences were obtained using the SQK-LSK109 reaction kit on an ONT Gridion instrument. Libraries were prepared following the manufacturer’s protocol. One R9.4.1 flow cell each was used for each specimen. Basecalling was performed with Guppy (v3.30 for *B. vancouverensis*, v.3.0.3 for other taxa) using default system settings. ONT reads for each species were concatenated into single fastq files and cleaned of adapters and chimeric reads using Porechop (https://github.com/rrwick/Porechop), including the–discard_middle flag. Short read sequence data (all 150 bp paired end reads) were generated by Illumina sequencing. Whole genome library preparation and sequencing for the *B. vosnesenskii* male (only JDL3184 was used for Illumina) was performed by HudsonAlpha Institute for Biotechnology (Huntsville, AL) on an Illumina Hiseq X, and data for *B. bifarius* and *B.vancouverensis* were generated by Psomagen, Inc (Rockville, MD) using the Illumina NovaSeq6000 S4 platform.

### Assembly

Preliminary assemblies indicated that reads included common bee symbionts and other bacteria, so we attempted to eliminate many such contaminants by preprocessing long and short read data sets using the bbtools program bbduk (Bushnell 2020). Decontamination used k-mer filtering (k = 29, although preliminary runs suggested altering this parameter made little difference in filtering results) against a reference dataset. This included a default set of contaminant reference fasta files provided with bbtools and supplemented with common bumble bee symbionts and other bacterial genomes detected as possible contaminants in preliminary assemblies, all downloaded from NCBI GenBank (Table S1). We filtered for human contaminants by mapping to a masked hg19 version of the human genome as a reference with the bbtools program bbmap, following recommended protocols in the manual (human contamination was minimal with <0.0001% of reads removed in all data sets).

The cleaned long and short reads were assembled using the MaSuRCA hybrid assembler (Zimin *et al.* 2013) with default parameters except for the Jellyfish estimate, which was set at 2.5 × 10^10^ (estimated genome size of ∼250 Mb, based on the closely related *Bombus impatiens* genome length (Hines 2008, Sadd *et al.* 2015), multiplied by an estimated coverage of 100x). MaSuRCA v3.3.1 was used for *Bombus vosnesenskii* and version 3.3.5 was used for other taxa.

We evaluated initial assemblies for contaminants using BlobTools v1.0 (Laetsch *et al.* 2017) to identify possible contaminants in the draft assembly based on scaffold taxonomic assignment (family level), sequencing coverage, and GC content. To prepare data for BlobTools, the Illumina and ONT reads were aligned to the draft genomes with BWA v0.7.15-r1140 (Li and Durbin 2009) and minimap2 v2.10 (Li 2018), respectively, and resulting files were sorted in SAMtools v1.10 (Li *et al.* 2009). A reference database for taxonomic assignment of scaffolds was created with blastn v2.9.0 using the NCBI nt database (downloaded on Feb. 18, 2019), with settings following the BlobTools manual (https://blobtools.readme.io/docs/taxonomy-file). Reads not mapping to scaffolds with a BLAST assignment to Hymenoptera were filtered out of the datasets. Remaining reads were used to run a second round of assembly with MaSuRCA, and then re-checked with BlobTools. As a final step, we filtered likely mitochondrial contaminants or other artifacts of assembly by excluding residual scaffolds < 10kb, matching families not within the order Hymenoptera, or with GC < 25%.

The genome assemblies were polished using PILON v1.23 (Walker *et al.* 2014), which uses the accurate Illumina data to correct for small base pair errors and small indels. Illumina reads were mapped to second-round assemblies with BWA and sorted with SAMtools. PILON was run with default parameters and repeated for a total of four sequential rounds of polishing.

### Quality assessment and species verification

Basic assembly statistics (genome length, number of scaffolds, GC content, and N50) were generated using QUAST v5.0 (Gurevich *et al.* 2013). We expected assemblies to be ∼250 Mb in size with a 38% GC content based on other *Bombus* genomes (Sadd *et al.* 2015; Kent *et al.* 2018). We examine genome completeness using BUSCO v4 (Simão *et al.* 2015) to search for orthologous genes using the OrthoDB v.10 (Kriventseva *et al.* 2019) Hymenopteran dataset (n = 5,991 genes) and using the bombus_impateins1 AUGUSTUS (Stanke and Morgenstern 2005) species parameter to optimize gene prediction. BUSCO genes detected in the assemblies were classified as single copy, duplicated, or fragmented. In the case of single or duplicated genes, this indicates that the gene is present and within 95% of its expected length, whereas fragmented genes fall outside of this limit (Simão *et al.* 2015). We perform basic comparisons of assembly statistics against two existing *Bombus* genomes (Sadd *et al.* 2015): *Bombus impatiens* assembly version 2.2 (BIMP_2.2 Assembly Accession GCF_000188095.3) and *Bombus terrestris* assembly version 1.0 (Bter_1.0 Assembly Accession GCF_000214255.1).

Finally, using the completed assemblies, we tested that genomes for *B. bifarius* and *B. vancouverensis*, two sister species in a morphologically cryptic complex (Ghisbain *et al.* 2020), were representative of diversity for their respective lineages. To confirm that our new genomes reflect these newly delimited species, we examined two nuclear genes (*serrate RNA effector* and *sodium/potassium-exchanging ATPase subunit alpha*) that were previously determined to produce diagnostic haplotypes for *B. bifarius* and *B. vancouverensis* and were employed for species delimitation in samples from throughout the *B. bifarius* – *B. vancouverensis* range (Ghisbain *et al.* 2020). We used blast to identify the relevant regions from the assemblies before aligning with GenBank sequences for the two species (NCBI PopSet 1803131478 for the RNA effector; PopSet 1803131398 for the ATPase) and generated a neighbor-joining distance tree (Jukes Cantor model) in Geneious Prime 2020.1.1 (Biomatters, Aukland NZ).

### Annotation

Genomes were submitted to NCBI RefSeq (Rajput *et al.* 2019) for annotation using the Eukaryotic Genome Annotation pipeline v8.4. This method has been used in other *Bombus* genomes (www.ncbi.nlm.nih.gov/genome/annotation_euk/all/), and thus provides standardization in the annotation methodology among members of the genus. For the annotations, in addition to transcript resources already on GenBank, we provided available RNAseq data generated from several individuals for each species from other prior and ongoing studies (see *Data Availability* below).

### Synteny analysis

We assessed synteny with the D-GENIES web-based software (Cabanettes and Klopp 2018), using minimap2 for alignment. We visualized synteny between our novel genomes and the previously assembled *Bombus terrestris* v1.0 genome. We use *B. terrestris*, which is more divergent from focal taxa than *B. impatiens* (∼18-20 million years ago; Hines 2008) but is a near-complete assembly with 18 linkage groups corresponding to the 18 bumble bee chromosomes (Owen *et al.* 1995; Stolle *et al.* 2011; Sadd *et al.* 2015). We restricted analyses to larger scaffolds (>100kb) for clearer visualization. To illustrate an example of one utility for these new assemblies, we performed a more detailed analysis of synteny for one scaffold which contains a high density of SNPs previously associated with signatures of selection relating to color pattern and environmental adaptation (NT_176739.1 in *B. impatiens*), especially in the region of the *Xanthine dehydrogenase/oxidase-like* gene in *B. bifarius* and *B. vancouverensis* (Pimsler *et al.* 2017; Ghisbain *et al.* 2020; Jackson *et al.* 2020). We had previously hypothesized that the outlier behavior across this region could involve a large-scale structural mutation (Pimsler *et al.* 2017). We examined synteny across homologous scaffolds for our new genomes (identified with blastn) and *B. impatiens* NT_176739.1 using the MAUVE (Darling *et al.* 2004) plug-in for Geneious to align scaffolds and compute co-linear blocks.

### Data availability

All raw data used for assembly (Illumina and ONT) and annotation (Illumina RNAseq) are available on the NCBI Sequence Read Archive (www.ncbi.nlm.nih.gov/sra/) as BioProjects PRJNA591177 for *Bombus bifarius*, PRJNA611633 for *B. vancouverensis*, and PRJNA611634 for *B. vosnesenskii*. RNAseq data supplied for annotation include BioProject PRJNA614946 for *B. bifarius* and *B. vancouverensis* and samples from PRJNA612513 for *B. vosnesenskii*. Whole genome shotgun projects have been deposited on GenBank under accessions JAAQOX000000000.1 for *B. bifarius*, JAAQRE000000000.1 for *B. vancouverensis*, and JAAQVK000000000.1 for *B. vosnesenskii*. Annotations described are NCBI *Bombus bifarius* Annotation Release 100, NCBI *Bombus vancouverensis nearcticus* Annotation Release 100, and NCBI *Bombus vosnesenskii* Annotation Release 100. Supplemental Information (Table S1) is available at figshare: https://doi.org/10.25387/g3.12181026.

## Results and Discussion

### Sequence data summary

Raw output from ONT sequencing yielded 1.72 × 10^6^ long reads (6.35 Gb; read N50 = 6.01 kb) for *B. bifarius*, 2.58 × 10^6^ reads (10.43 Gb; read N50 = 6.79 kb) for *B. vancouverensis*, and 2.08 × 10^6^ reads (9.00 Gb; read N50 = 7.60 kb) for *B. vosnesenskii*, representing an estimated coverage between ∼25x and ∼42x based on the assumed genome size of ∼250 Mb. Illumina sequencing yielded 9.00 × 10^7^ read pairs (26.98 Gb) for *B. bifarius*, 8.97 × 10^7^ pairs (26.92 Gb) for *B. vancouverensis*, and 8.90 × 10^7^ pairs (26.72 Gb) for *B. vosnesenskii*, producing an estimated short read coverage of ∼100x (Table 2). All data sets contained a small degree of sequence from bee symbionts or other contaminants removed during pre-assembly filtering (Table 2; Table S1).

**Table 2:** Sequencing statistics. Number of reads (read pairs for Illumina) at each stage in data filtering, including raw data, bacteria-filtered first-assembly data, and filtered data passed to the final second-round assembly. Estimated coverage is based on the number of sequence bases provided to the final assembly and an assumed genome size similar to *B. impatiens* (245.9 Mb)

Species	Sequencing	No. raw reads (No. bases)	No. reads first assembly (No. bases)	No. reads second assembly (No. bases)	Estimated coverage
*B. bifarius*	Illumina	9.00x10^7^ (26.98 Gb)	8.98x10^7^ (26.94 Gb)	8.49x10^7^ (25.48 Gb)	103.6x
	ONT	1.72x10^6^ (6.35 Gb)	1.63x10^6^ (5.91 Gb)	1.46x10^6^ (5.57 Gb)	22.7x
*B. vancouverensis*	Illumina	8.97x10^7^ (26.92 Gb)	8.96x10^7^ (26.86 Gb)	8.06x10^7^ (24.18 Gb)	98.3x
	ONT	2.58x10^6^ (10.43 GB)	2.53x10^6^ (9.92 Gb)	2.27x10^6^ (9.19 Gb)	37.4x
*B. vosnesenskii*	Illumina	8.90x10^7^ (26.72 Gb)	8.88x10^7^ (26.66 Gb)	8.20x10^7^ (24.62 Gb)	100.1x
	ONT	2.08x10^6^ (9.00 Gb)	2.02x10^6^ (8.55 Gb)	1.72x10^6^ (7.84 Gb)	31.9x

### Assembly quality

**Figure 2:**
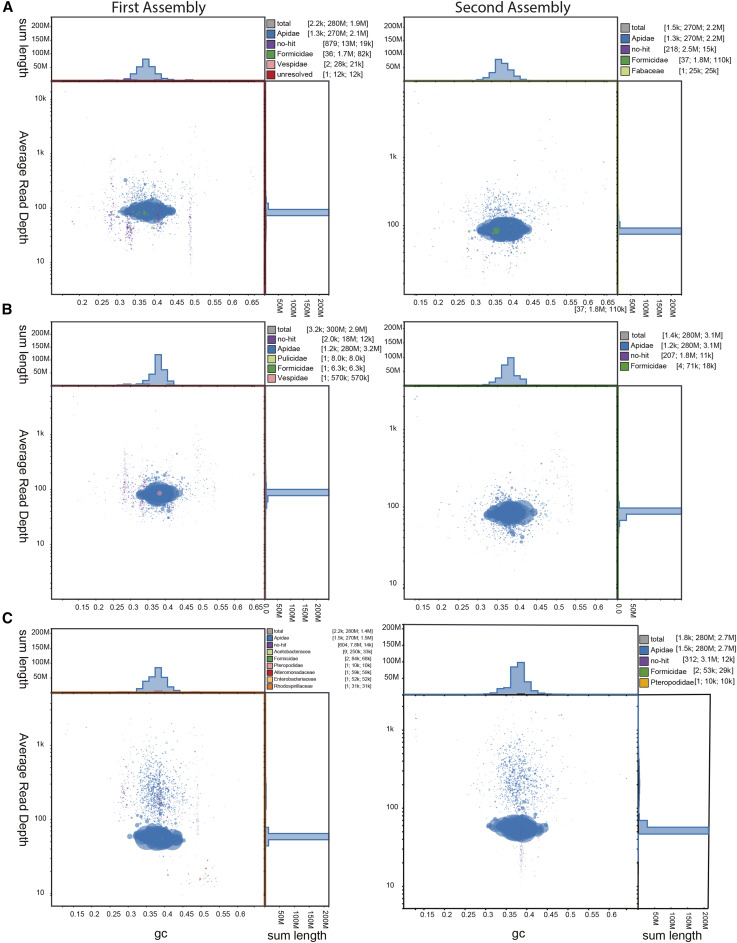
Blob plots (from Illumina data) showing read depth of coverage, GC content, and size for each scaffold after first (left hand column) and second round (right hand column) MaSuRCA assemblies for A) *Bombus bifarius*, B) *Bombus vancouverensis*, C) *Bombus vosnesenskii*. Size of the blob corresponds to size of the scaffold and color corresponds to taxonomic assignment of BLAST (blue = Apidae). Inset statistics for each panel refer to [scaffold count, sum length, N50] associated with BLAST assignments to each taxonomic group. The top and right histograms indicate the total length of scaffolds at a given GC content or average read depth, respectively. Qualitatively similar plots were produced using the ONT data.

After the preliminary assembly round, more than 95% of the scaffolds had BLAST hits to Apidae and other Hymenoptera (Figure 2). Some scaffolds represented bacterial or other genomes not removed during the pre-assembly decontamination and many small scaffolds did not match any taxonomic group. Such scaffolds represented less than 5% of the total genome assemblies, however (< 0.10 Gb for both Illumina and ONT reads; Figure 2, Table 2). Following filtering with BlobTools, the second-round assemblies had many fewer contaminants, with <0.01% of scaffolds having non-Hymenopteran BLAST hits (Figure 2).

**Figure 3:**
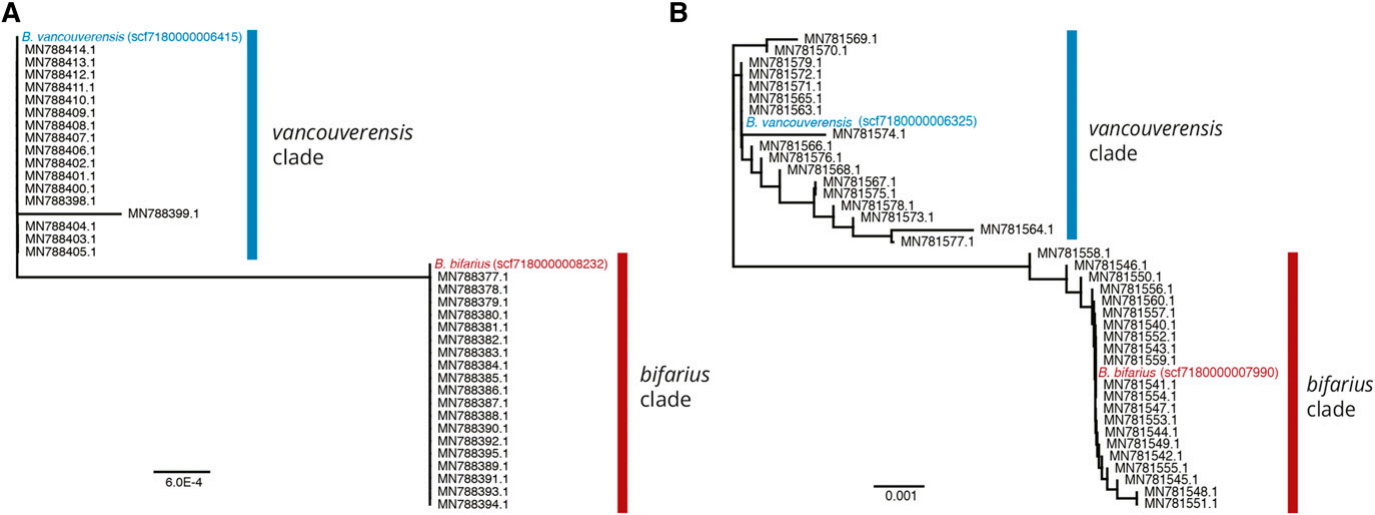
Confirming sample identity for the *B. bifarius* and *B. vancouverensis* genomes. A-B) Neighbor joining distance trees for the A) *serrate RNA effector* and B) *sodium/potassium transporting ATPase subunit alpha* genes from *Bombus bifarius* and *Bombus vancouverensis* assemblies aligned to GenBank accessions (tip labels on tree) originally used for delimitation of these sister species (from NCBI PopSet 1803131478 and 1803131398; Ghisbain *et al.* 2020).

The final assembly lengths (Table 3) were slightly larger than the closely related *Bombus* genomes (Sadd *et al.*, 2015) (266.8 Mb in 1,249 scaffolds for *B. bifarius*, 282.1 Mb in 1,162 scaffolds for *B. vancouverensis*, and 275.6 Mb in 1,429 scaffolds for *B. vosnesenskii*), but GC contents were similar to other *Bombus* at ∼38% (Sadd *et al.* 2015) (Figure 2, Table 3). The assemblies are all highly complete, with N50’s of 2.2 - 3.06 and BUSCO scores of >98% for the 5,991 genes in the OrthoDB v.10 Hymenoptera lineage dataset. Most of these (>97.7%) were single copy, with <1% of the reference gene set duplicated or fragmented, and ∼1–1.3% missing (Figure 2, Table 3). Although haploid males allow sequencing of phased haplotypes, there was not a clear influence of ploidy of starting material (haploid male for *B. bifarius*, diploid worker for *B. vancouverensis*, two haploid males for *B. vosnesenskii*) on the final assembly quality in terms of N50 or BUSCO scores (Table 3). Finally, we also confirm that sequences from *B. bifarius* and *B. vancouverensis* assemblies for two genes previously employed as diagnostic evidence for species delimitation (Ghisbain, *et al.* 2020) were representative of the diversity found in each species (Figure 3A, B).

**Table 3:** Assembly statistics and BUSCO analyses for the three focal species genomes in comparison to other *Bombus* genomes

	Assembly statistics				BUSCO results*^a^*		
Species	Length (Mb)	N50 (Mb)	No. scaffolds	GC %	Complete [single, duplicated]	Fragmented	Missing
*B. bifarius*	266.8	2.20	1,249	37.96	98.1% [97.7%,0.4%]	0.6%	1.3%
*B. vancouverensis*	282.1	3.06	1,162	38.02	98.4% [97.9%,0.5%]	0.6%	1.0%
*B. vosnesenskii*	275.6	2.83	1,429	37.93	98.2% [98.0%, 0.2%]	0.6%	1.2%
*B. terrestris* v.1.0*^b^*	248.7	12.9	5,609	37.51	96.9% [96.7%, 0.2%]	1.5%	1.6%
*B. impatiens* v.2.2*^c^*	245.9	1.41	2,506	37.76	98.3% [98.1%, 0.2%]	0.7%	1.0%

a BUSCO analysis run using the OrthoDB v.10, Hymenoptera dataset containing 5,991 genes.

b *Bombus terrestris* genome assembly version: Bter_1.0.

c *Bombus impatiens* genome assembly version: BIMP_2.2.

### Annotation

Gene predictions from the NCBI Eukaryotic Annotation Pipeline resulted in 13,325 – 13,687 genes for the new genomes, with statistics for numbers of genes, transcripts, and other features largely consistent across assemblies and with previous *Bombus* assemblies (Table 4).

**Table 4:** Annotation statistics from NCBI Eukaryotic genome annotation pipeline for the three focal species genomes in comparison to other *Bombus* genomes. In the case of the three focal genomes, all are on annotation release 100 whereas *B. impatiens* and *B. terrestris* are on 103 and 102, respectively. Details on data used for annotation and comparative statistics are available at the NCBI links given in footnotes a-c

	*B. bifarius**^a^*	*B. vancouverensis**^b^*	*B. vosnesenskii**^c^*	*B. impatiens**^d^*	*B. terrestris**^e^*
**Genes: total**	**13,325**	**13,687**	**13,527**	**13,161**	**11,083**
Protein coding genes	11,148	11,338	11,184	10,632	10,400
Non-coding genes	1,653	1,802	1,789	2,293	607
Pseudogenes	524	547	554	236	76
**mRNA: total**	**23,896**	**24,385**	**24,067**	**24,471**	**20,321**
**Non-coding RNA: total**	**2,731**	**2,964**	**2,974**	**3,542**	**1,428**

a https://www.ncbi.nlm.nih.gov/genome/annotation_euk/Bombus_bifarius/100/

b https://www.ncbi.nlm.nih.gov/genome/annotation_euk/Bombus_vancouverensis_nearcticus/100/

c https://www.ncbi.nlm.nih.gov/genome/annotation_euk/Bombus_vosnesenskii/100/

d https://www.ncbi.nlm.nih.gov/genome/annotation_euk/Bombus_impatiens/103/

e https://www.ncbi.nlm.nih.gov/genome/annotation_euk/Bombus_terrestris/102/

### Genome comparisons

**Figure 4:**
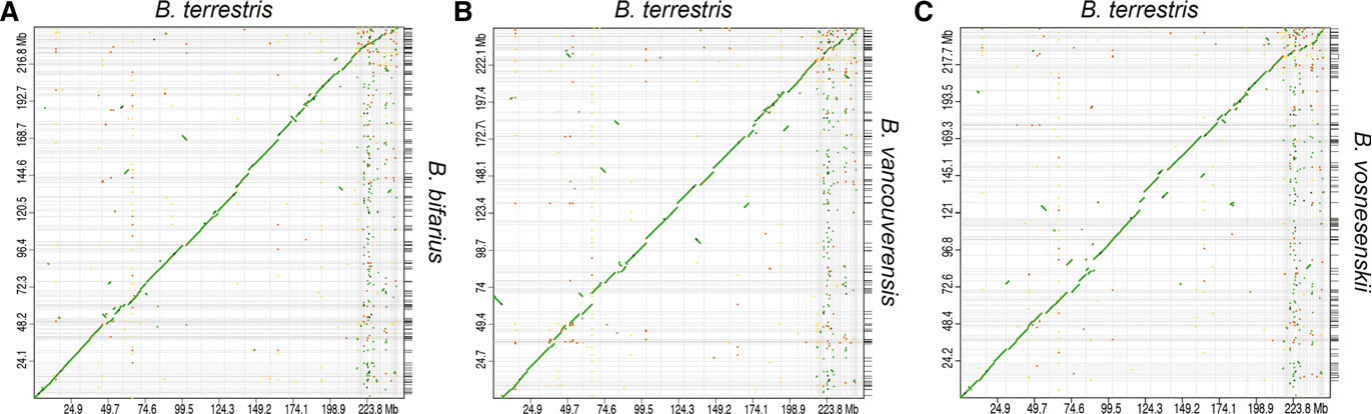
D-GENIES dot plots (using Minimap2 aligner) indicating collinearity of scaffolds (>100 kb in length) with the *Bombus terrestris* genome for A) *Bombus bifarius*, B) *Bombus vancouverensis*, and C) *Bombus vosnesenskii*.

**Figure 5:**
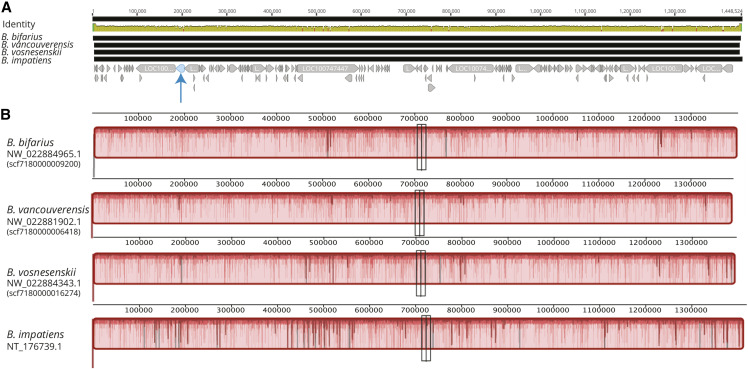
Analysis of *de novo* genomes in a region previously identified as a candidate target of selection in these lineages. A) MAUVE alignment for a focal scaffold of interest in the new genomes and in *B. impatiens*, which has repeatedly shown evidence of local adaptation in prior studies using *B. impatiens* as a reference genome. The bottom track indicates gene IDs from *B. impatiens*, with the arrow pointing to a previously discovered gene of interest in the region (LOC100741462, *Xanthine dehydrogenase/oxidase*-like; Pimsler *et al.* 2017); B) MAUVE produced a single collinear orientation block across species suggesting no major structural rearrangements in the region.

We analyzed the synteny of genomes with respect to the *B. terrestris* genome assembly. The new genomes were highly collinear with the 18 *B. terrestris* linkage groups (Figure 4), although several rearrangements were apparent in each species. Such patterns may reflect some true rearrangements between the focal species assemblies and *B. terrestris* genome that have occurred over 18-20 million years of divergence but may also be the result of a small number of assembly artifacts. Our focused MAUVE analysis of the region containing a high density of putatively adaptive loci from prior studies (including a possible color-associated gene *Xanthine dehydrogenase/oxidase*-like; Pimsler *et al.* 2017; Ghisbain *et al.* 2020; Jackson *et al.* 2020) revealed a single collinear block (Figure 5A, B). This suggests no major rearrangements in the new genomes with respect to the *B. impatiens* genome previously used as a reference genome for population genomics in these species. Thus, major structural mutations may not explain unusual patterns of variation in the region, although more detailed intraspecific analyses will be necessary to fully rule out a role for segregating rearrangements in local adaptation.

In conclusion, the assemblies for each of the three focal species were highly complete, demonstrating how Illumina and long read ONT sequences can be used to quickly and inexpensively assemble high quality *de novo* genomes. The newly assembled genomes are intact, with 90% of each assembly contained in scaffolds ≥ 50kb and N50’s > 2.2 Mb, and compare well to the other published bumble bee genomes in terms of overall size, GC content, BUSCO scores, and numbers of predicted genes and other features. Further, the results support previous observations that bumble bee genome structure tends to be conserved over relatively deep timescales, with large-scale synteny over ∼18-20 million years of separation (Sadd *et al.* 2015). We expect these new references will quickly become useful for bumble bee biologists. For example, the species included here have all recently been studied with genomic data to detect the influence of climate and landscape composition on dispersal and local adaptation (Jackson *et al.* 2018; Jackson *et al.* 2020; Mola *et al.* 2020), however, such studies have largely been limited to genome-reduction methods (*e.g.*, RAD-seq) and have required cross-species mapping to available reference genomes. These new assemblies will open the door for more extensive whole genome resequencing to uncover unique genomic variation that may be shaped by environmental conditions for each species (Fuentes-Pardo and Ruzzante 2017). These assemblies will also provide additional data points for multi-genome comparative studies in *Bombus* and other bees (*e.g.*, Kapheim *et al.* 2015; Lin *et al.* 2019). These new resources should thus prove valuable for researchers looking to answer questions relating to diverse aspects of bumble bee evolutionary biology.
